# Lymphoma Immunotherapy: Current Status

**DOI:** 10.3389/fimmu.2015.00448

**Published:** 2015-09-01

**Authors:** Roberta Zappasodi, Filippo de Braud, Massimo Di Nicola

**Affiliations:** ^1^Ludwig Collaborative and Swim Across America Laboratory, Memorial Sloan Kettering Cancer Center, New York, NY, USA; ^2^Unit of Medical Oncology, Fondazione IRCCS Istituto Nazionale dei Tumori, Milan, Italy; ^3^Unit of Immunotherapy and Anticancer Innovative Therapeutics, Fondazione IRCCS Istituto Nazionale dei Tumori, Milan, Italy

**Keywords:** B-cell lymphoma, immunotherapy, anticancer vaccines, tumor-associated antigens, dendritic cells, adaptive immune response

## Abstract

The rationale to treat lymphomas with immunotherapy comes from long-standing evidence on their distinctive immune responsiveness. Indolent B-cell non-Hodgkin lymphomas, in particular, establish key interactions with the immune microenvironment to ensure prosurvival signals and prevent antitumor immune activation. However, reports of spontaneous regressions indicate that, under certain circumstances, patients develop therapeutic antitumor immunity. Several immunotherapeutic approaches have been thus developed to boost these effects in all patients. To date, targeting CD20 on malignant B cells with the antibody rituximab has been the most clinically effective strategy. However, relapse and resistance prevent to cure approximately half of B-NHL patients, underscoring the need of more effective therapies. The recognition of B-cell receptor variable regions as B-NHL unique antigens promoted the development of specific vaccines to immunize patients against their own tumor. Despite initial promising results, this strategy has not yet demonstrated a sufficient clinical benefit to reach the regulatory approval. Several novel agents are now available to stimulate immune effector functions or counteract immunosuppressive mechanisms, such as engineered antitumor T cells, co-stimulatory receptor agonist, and immune checkpoint-blocking antibodies. Thus, multiple elements can now be exploited in more effective combinations to break the barriers for the induction of anti-lymphoma immunity.

## Introduction

Lymphomas are a clinically and biologically heterogeneous group of malignancies that arise from mature T- or B-lymphocytes in secondary lymphoid organs. Hodgkin’s lymphomas (HLs) account for ~10% of all lymphomas and comprise two major disease categories based on their clinical and histological characteristics: classical HLs, which represent the majority of the cases, and nodular lymphocyte predominant HLs. Non-Hodgkin lymphomas (NHLs) instead are much more frequent diseases, representing the fifth most common cancer in the United States. Their incidence has progressively increased in the past three decades for non-completely certain reasons (1). About 85% of NHLs are of B-cell origin (B-NHLs) and includes a wide spectrum of malignancies with different clinical and biological courses, ranging from indolent [such as chronic lymphocytic leukemia/small lymphocytic lymphoma (CLL), follicular lymphoma (FL), and marginal-zone lymphoma (MZL)] to aggressive [such as diffuse large B-cell lymphoma (DLBCL), Burkitt’s lymphoma (BL), and mantle cell lymphoma (MCL)].

These tumors, in particular the aggressive forms, are highly sensitive to both chemotherapy and radiotherapy (2); however, relapse and resistance prevent the ultimate goal of achieving a cure in all patients. In the last few decades, the introduction of improved chemotherapy regimens, monoclonal antibodies (mAbs), radioimmunotherapy, and targeted therapies against pro-lymphoma pathways have provided significant advances in the management of these patients, in particular those with B-NHLs. The chimeric anti-CD20 mAb rituximab has been the most valuable addition to the B-NHL treatment armamentarium. Its combination with poly-chemotherapy still represents the standard therapy for both indolent and aggressive B-cell lymphomas (3, 4). However, difficulties in the management of relapse and resistance to rituximab (5, 6) and the late toxicities associated with its administration (7) still pose significant challenges. Alternative approaches are thus continuously sought to ameliorate the management and the clinical outcome of the many patients that become resistant to rituximab.

In the past 20 years, the understanding of the molecular basis of B-cell lymphomagenesis and the role of the lymphoma microenvironment has significantly progressed, thus underscoring multiple novel rational therapeutic modalities for B-cell malignancies.

B-cell maturation is dictated by a series of steps that drive the development of a functional B-cell receptor (BCR) with the same antigen specificity as the secreted Abs that B cells will eventually produce. A BCR is composed of two clonally variable antigen-binding chains (heavy and light chains) codified by several different gene segments (V, variable; D, diversity; J, joining; C, constant), which need to be properly rearranged to produce a functional antigen-binding receptor. This occurs via an error-prone process involving the combinatorial rearrangement of the V, D, and J gene segments in the heavy (H) chain locus and the V and J gene segments in the light (L) chain loci. Mature (naïve) B cells carry a BCR composed of two identical heavy chain and two identical light-chain immunoglobulin (Ig) polypeptides covalently linked (8). Antigen recognition by naïve B cells favors their recruitment into lymphoid follicles where they undergo somatic hypermutation of V genes, to increase the affinity for the targeted antigenic epitopes, and class switch recombination at the IgH locus, for the production of different classes of Ab (from IgM to IgG, IgA, or IgE). These processes form the germinal center (GC) reactions, whereby new B-cell clones expressing Abs with improved antigen specificity and suitable class are positively selected by receiving the proper survival signals from follicular dendritic cells (DCs) presenting the pathogenic antigens and helper T cells (9). If, on one hand, these events are required to increase the probability of generating a specific B-cell response able to clear infecting pathogens, on the other, they pose at risk of developing oncogenic mutations. DNA rearrangement, induction of somatic mutation, and provision of anti-apoptotic/pro-survival signals from the microenvironment during the B-cell maturation process may all favor the generation of a malignant B-cell clone if not tightly regulated. Reciprocal chromosomal translocations involving one of the Ig loci and a proto-oncogene, which may occur as by-products of the extensive DNA rearrangement during the GC reactions, constitute the hallmarks, and thus diagnostic markers, of many types of B-cell lymphoma (10, 11) (Table 1).

**Table 1:** **B-cell lymphoma classification**.

Lymphoma	Frequency among lymphoma (%)	Proposed cellular origin	Chromosome translocation (frequency)	Tumor-suppressor gene mutation (frequency)	Viruses (frequency)	Other alterations (frequency)
cHL	9	GC B cells	–	SOCS1 (40), NFKBIA and NFKBIE (10–20), A20 (40)	EBV (40)	Mutation of multiple oncogenes, including REL (30), JAK2 (20), NIK (25)
NLPHL	1	GC B cells	–		EBV	
B-CLL	7	CD5+ small memory, naive, or marginal-zone B cells	–	ATM (30), TP53 (15)	–	Deletion on 13q14 (60)
MCL	5	CD5+ mantle-zone B cells	CCND1-IgH (95)	ATM (40)	–	Deletion on 13q14 (50–70)
FL	20	GC B cells	BCL2-IgH (90)	–	–	–
MALT	7	Marginal-zone B cells	API2-MALT1 (30), BCL10-IgH (5), MALT1-IgH (15–20), FOXP1-IgH (10)	CD95 (5–80)	Indirect role of Helicobacter Pylori in gastric MALT lymphomas	–
MZL	2	Marginal-zone or monocytoid B cells	–	–	–	–
Splenic MZL	1	Small IgD+ naive marginal-zone B cells	–	–	–	Deletion on 7q22–36 (40)
BL	2	GC B cells	MYC-IgH or MYC-IgL (100)	TP53 (40), RB (20–80)	EBV (endemic, 95; sporadic, 30)	–
DLBCL	30–40	Post-GC B cells	BCL6–various (35) BCL2-IgH (15–30) MYC-IgH or MYC-IgL (15)	CD95 (10–20), ATM (15), TP53 (25)	–	Aberrant hypermutation of multiple proto-oncogenes (50)
Primary mediastinal B-cell lymphoma	2	Thymic B cells	–	SOCS1 (40)	–	Mutation of multiple ­proto-oncogenes (40)
Post-transplant lymphoma	<1	GC B cells	–	–	EBV (90)	–
Primary effusion lymphoma	<0.5	(Post) GC B cells	–	–	HHV8 (95), EBV (70)	–
LPL; Waldenstrom’s disease	1	(Post) GC B cells	PAX5-IgH (50)	–	–	–

*cHL, classical Hodgkin’s lymphoma; NLPHL, nodular lymphocyte predominant Hodgkin’s lymphoma; B-CLL, B-cell chronic lymphocytic leukemia; MCL, mantle-cell lymphoma; FL, follicular lymphoma; MALT, mucosa associated lymphatic tissue lymphoma; MZL, marginal zone lymphoma; BL, Burkitt’s lymphoma; DLBCL, diffuse large B-cell lymphoma; LPL, lymphoplasmacytic lymphoma; GC, germinal center*.

Mutations in pro-apoptotic genes (CD95), tumor-suppressor genes (TP53, PTEN), BCR downstream signaling pathways (CD79B/A, IκBα, CARD11, API2–MALT1 translocation) and other oncogenes (EZH2, Jak2, genomic amplifications REL) are associated to specific subtypes of B-cell lymphomas, indicating a role of these events in their pathogenesis (Table 1).

Finally, viruses may also be involved in lymphoma transformation, in particular Epstein–Barr virus (EBV), Kaposi’s sarcoma herpesvirus (KSHV), human immunodeficiency virus type 1 (HIV-1), and human hepatitis C virus (HCV). They can directly infect and transform B or T cells (EBV and KSHV), or induce lymphocyte transformation as a consequence of chronic inflammation (HIV, EBV, and hepatitis viruses), or, more indirectly, promote the onset of neoplastic clones by causing immunodeficiency (HIV-1) (12). The most obvious example in this regard is EBV, which is found in nearly all the endemic BLs and in many post-transplant and primary effusion lymphomas (13, 14).

The possibility to interfere with ongenic pathways activated in the different subtypes of B-cell lymphomas has been an area of intense investigation, with two molecular inhibitors targeting bruton tyrosine kinase (ibrutininb) (15–17) or PI3K (idelalisib) (18) receiving the FDA approval for the treatment of relapsed/resistant B-NHLs in the last 2 years. However, since these therapies target oncogenic events associated to specific molecular lymphoma subtypes, they are unlikely to be available for all rituximab-resistant patients, and imply the requirement of an up-front extensive molecular characterization. In addition, being directed against a single molecular target, these drugs may induce the selection of resistant clones. This indicates the need to integrate anti-lymphoma treatments in multicombinatorial therapeutic approaches, which employ different strategies to reach the desired improvement in clinical benefit.

Immunotherapy seems one of the best candidates because of the easy accessibility of lymphomas by the immune system as they grow in secondary lymphoid organs and the availability of unique targetable tumor-specific antigens. The major advantage of immunotherapy is the possibility to induce an adaptive immune response against the tumor, with the potential to generate a long-lasting immunological memory able to prevent further relapses. Carrying the same BCR on the surface, B-cell lymphomas are distinguished by the unique antigenic determinants of BCR hypervariable regions, termed idiotype (Id), which constitutes a prototype immunotherapeutic target to specifically redirect immune responses against the malignant clone. The clonotypic Id of B-cell malignancies was indeed the first identified tumor-specific antigen able to elicit a T-cell response (19, 20). The crucial interactions between lymphoma cells and the immune microenvironment for their maintenance and progression, in particular in the case of HL and indolent B-NHLs (21), have underscored other potential immunotherapeutic targets. Tumor-infiltrating immune cells, including T lymphocytes, macrophages and DCs, can provide survival signals for malignant B cells (22, 23). As an example, FL growth strictly depends on stromal cells, such as follicular DCs, which provide anti-apoptotic signals through CD40 (24, 25). On the other hand, T regulatory cells (Tregs) (26–28) and immunosuppressive lymphoma-associated macrophages (29–31) can contribute to lymphoma growth by dampening the immune system attack. Therefore, acting on the immune microenvironment can also be exploited as a rational anti-lymphoma immunotherapeutic treatment.

The following sections review the most important and recent advances in anti-lymphoma immunotherapy, with a particular focus on strategies exploiting the T-cell arm of the immune response against B-NHLs.

## Anti-Lymphoma Immunotherapy

Anticancer immunotherapy is aimed at eradicating tumor cells by conferring either a passive or an active specific immunity with less toxic effects than using conventional anticancer agents. Passive immunotherapy is meant to supply the immune response through the infusion of tumor-specific mAbs or cytotoxic T cells (CTLs), with the major limitation that it may be short-lived. Active immunotherapy instead is thought to stimulate an endogenous immune response to clear neoplastic cells and induce a specific immunological memory that controls disease recurrence, and thus represents an ideal immunotherapeutic modality. More recently, thanks to the development of immunomodulatory agents, a new area of immunotherapy has started to be explored with the aim to induce and/or sustain endogenous antitumor immune responses, providing substantial clinical results.

B-NHLs, in particular the indolent forms, represent one of the most suitable settings for immunotherapeutic interventions. They have long been regarded as highly immune sensitive diseases, based on the detection of lymphoma-specific CTLs in B-NHL patients (32) and reports of spontaneous regressions in 10–20% of the low-grade cases. Moreover, the course of indolent lymphomas leaves an optimal therapeutic window to study immunotherapy without affecting the standard of care of these patients, given that immunotherapy, relying on endogenous immune system functions, may require longer periods of time to induce a therapeutic effect.

In the last two decades, a number of immune-based treatments have been developed and tested in B-NHL patients. To date, the use of mAbs directed against B-NHL antigens has produced the most convincing results, with rituximab being the prototype example in this treatment category. The introduction of rituximab-based chemoimmunotherapy has improved the overall survival (OS) of indolent lymphoma patients, providing a change in the natural history of these diseases (33, 34). However, resistance to rituximab remains a problem (35) and more effective regimens are still needed. MAbs for new lymphoma targets as well as new generation Abs are thus being developed with the aim to further ameliorate patients’ outcome.

Patient-specific vaccines targeting the clonally derived Ig-Id protein or the whole antigenic tumor repertoires have been largely tested against B-NHLs, with certain degrees of success also in severely pretreated patients (36). Furthermore, on the basis of the high sensitivity of these diseases to graft-versus-tumor effects after allogeneic bone marrow transplantation/donor lymphocytes infusions (DLIs), adoptive transfer of tumor-specific CTLs has been also used in lymphoma patients (37, 38). Building upon these findings, lymphoma-specific chimeric antigen receptor (CAR)-engineered T cells are now being explored for the treatment of lymphoma patients with very promising results (39). Finally, the availability of immune checkpoint-blocking agents (40, 41) now allows the opportunity to counteract immune tolerant mechanisms, which are considered the major obstacle to the efficacy of anticancer immunotherapy, and to explore potentially more effective immunotherapeutic combinations against B-NHLs.

## Active Immunotherapy for B-Cell Lymphomas

**Figure 1:**
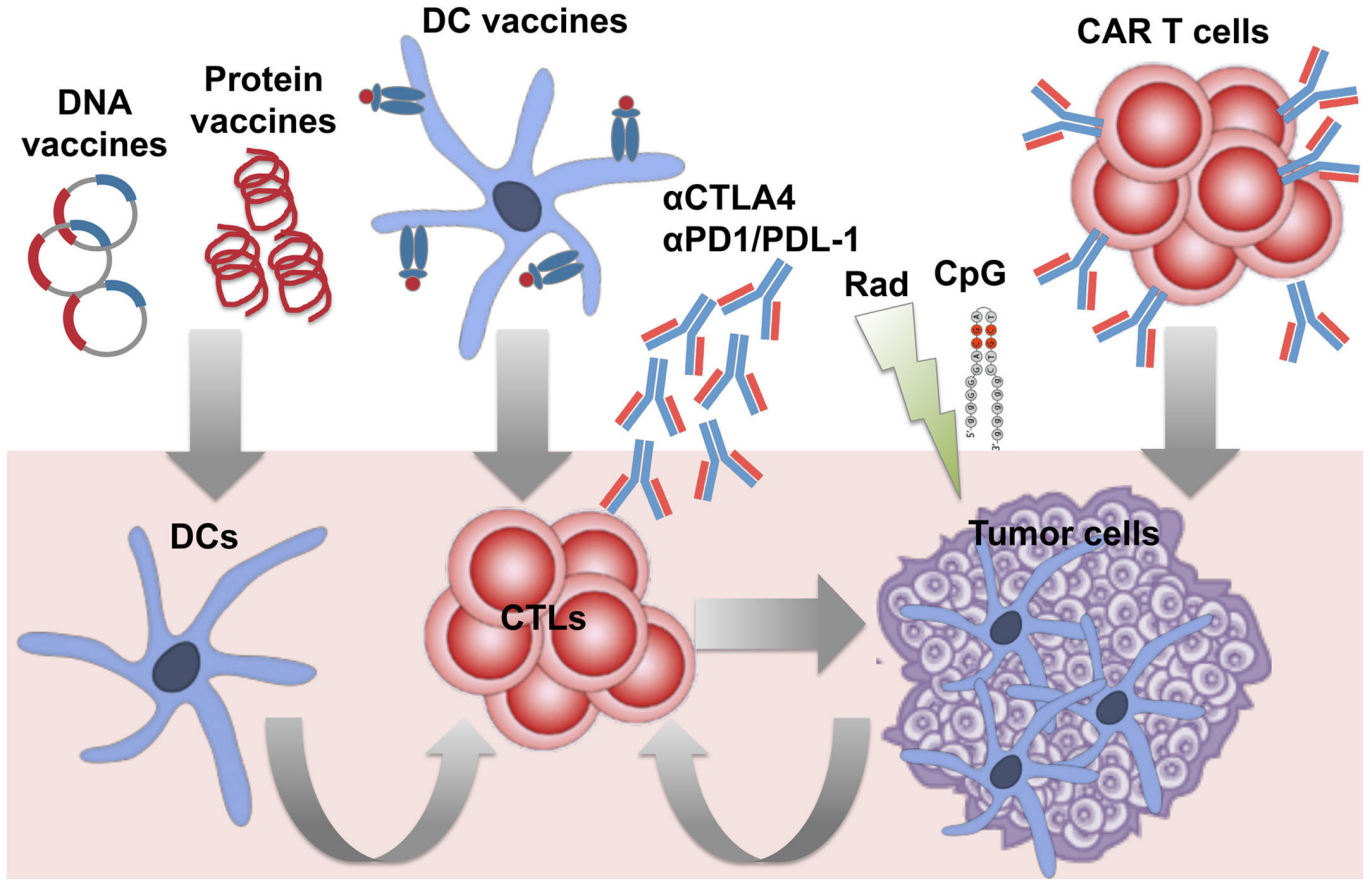
**Immunotherapeutic strategies under investigation against B-cell lymphomas**. Several approaches have been developed to induce therapeutic anti-lymphoma T-cell responses, by either targeting dendritic cells (DCs) *in vivo* or *ex vivo*, or adoptive transfer of specific cytotoxic T cells (CTLs), and/or appropriate modulation of T-cell functions *in vivo*. Active immunization with patient-specific Id proteins or DNA plasmids encoding for the Id have been exploited to target DCs *in vivo* and activate T cell against B-cell lymphomas. DCs optimally pulsed *ex vivo* with lymphoma antigens (Id or whole tumor antigens) have been employed as vaccines to improve the stimulation of specific T cells *in vivo*. To bypass *in vitro* manipulation, the strategy to induce *in vivo* immunogenic lymphoma cell death (with radiation therapy) and activation of DCs (with the TLR agonist CpG) has been studied to favor the occurrence of a vaccinal effect *in vivo* (*in situ* vaccination). To overcome the difficulties of generating endogenous T-cell responses able to eradicate tumors in pluritreated lymphoma patients, adoptive transfer of activated tumor-specific T cells (such as anti-lymphoma CAR-engineered T cells) has been also investigated. Finally, the availability of several immunomodulatory agents offers the opportunity to target the tumor immune microenvironment from multiple sides. Blocking Abs against the immune checkpoints PD-1 and CTLA-4 are among the first therapies in the pipeline to be tested with the aim to boost T-cell functions and counteract immunosuppression in lymphoma patients.

The availability of a tumor-specific antigen in B-NHLs enabled the development of specific vaccines. Id immunodominant peptides or the whole Id determinants have been extensively used to vaccinate patients as protein- or DNA-based vaccines or loaded into DCs (Figure 1) (36). Different types of carriers and immune adjuvants have been combined with these vaccines to potentiate the activation of an immune response against a self-antigen. As an alternative strategy to reduce the complexity of the production of patient-specific Id and widen the spectrum of targeted tumor-associated antigens (TAAs), vaccines based on the whole lymphoma proteome have been investigated. Whereas protein- and DNA-based vaccines are designed to target DCs *in vivo*, whole tumor cell antigens have been usually loaded into DCs *ex vivo*, with the advantage to select/generate the most suitable source of DCs able to efficiently present TAAs and activate an immune response *in vivo* upon injection (Figure 1).

## Protein-Based Vaccines

Anti-Id vaccines have used Id proteins produced by either somatic hybridization of tumor cells with a myeloma cell line (hybridoma), or recombinant technology, by cloning Ig genes into stable cell lines (36). The latter strategy is faster, taking 1 month, but in contrast to the hybridoma technology, the Id glycosylation pattern, and in turn immunogenicity, is strictly dependent on the origin of the cell line used (42). The capability of the Id vaccination to induce tumor protection was extensively demonstrated in plasmacytoma, myeloma, B-cell lymphoma, and leukemia preclinical models (36). Being *per se* a weakly immunogenic protein, the Id was conjugated to the carrier protein keyhole limpet hemocyanin (KLH) and co-administrated with low-dose granulocyte-macrophage colony-stimulating factor (GM-CSF). This strategy demonstrated to promote anti-Id B- and T-cell responses associated with therapeutic effects in animals with low tumor burden (36), and paved the way for the clinical evaluation of anti-Id vaccination.

Early-phase clinical studies were performed in indolent B-NHL patients in clinical remission after standard chemotherapy regimens, using Id proteins produced either by hybridoma or recombinant technology, conjugated with KLH and co-administered with low-dose GM-CFS or Syntex adjuvant formulation (43). These studies demonstrated the feasibility of producing patient-specific Id-vaccines, and the safety and efficacy of this strategy to induce anti-lymphoma immune responses, eventually associated with an improved clinical outcome (43). In line with the preclinical results, the co-administration of low-dose GM-CSF with Id-KLH showed to promote anti-Id T-cell responses and molecular remissions in patients with minimal residual disease after prednisone, doxorubicin, cyclophosphamide, and etoposide (PACE) induction therapy (44). In a following trial, anti-Id vaccination after cyclophosphamide, doxorubicin, vincristine, prednisone (CHOP)-like second-line induction therapy resulted in longer clinical remissions compared to those achieved in the same patients by the front-line standard therapy (45). Interestingly, patients mounting either an Ab or a T-cell anti-Id response after vaccination experienced the longest second complete remission, providing the first in-human evidence of the association between vaccine-specific immune responses and clinical efficacy. A more recent retrospective study demonstrated that achieving a complete response/complete response unconfirmed (CR/CRu) to induction chemotherapy and developing anti-Id Abs were two independent factors that each correlated with longer OS at 10 years after vaccination (46). This study included FL patients who received vaccines produced by either the hybridoma or recombinant technology in both mammalian cells and in tobacco plants. Interestingly, the probability of developing an anti-Id immunity was not influenced by the method of vaccine generation, although in patients vaccinated with hybridoma-derived Id, the rate of specific T-cell responses trended to be higher and the correlation between anti-Id Ab responses and OS resulted particularly significant (46). This is probably due to the presence of a more physiological glycosylation pattern in the hybridoma-derived Id, which may improve the immunogenicity of the Id. Given the critical role of the induction of anti-Id immune responses for the therapeutic efficacy of Id vaccination, two clinical trials with Id-KLH + GM-CSF explored the impact of B-cell depletion by rituximab as part of the induction therapy before vaccination. Importantly, they showed that, even if delayed, Id-specific Ab responses could be equally achieved, whereas the induction of antitumor T-cell immunity was not affected (47, 48). Remarkably, an improved time to progression (TTP) was reported for patients receiving vaccination after rituximab compared to the historical controls treated with rituximab alone, suggesting a potential clinical benefit of active immunotherapy also in the setting of B-cell recovery after rituximab therapy.

The feasibility, tolerability, and efficacy of Id vaccines demonstrated in early-stage clinical trials led to the initiation of three large-scale randomized phase-III studies aimed at demonstrating a clear-cut survival improvement in vaccinated patients. They tested either recombinant Id (MyVax, Genitope Corporation (49); FavId, Favrille) (50) or hybridoma-derived Id (BiovaxId, Biovest International Inc.) (51) in grades 1–3 FL patients that achieved at least disease stabilization (50), partial (49) or complete (51) remission after induction with a standard course of rituximab or cyclophosphamide, vincristine and prednisone (CVP) or PACE, respectively (Table 2).

**Table 2:** **Main features and interpretation of phase-III clinical trials with anti-Id vaccination**.

	Genitope	Favrille	NCI/Biovest
Vaccine	MyVax	FavId	BiovaxId
Patients	FL, untreated	FL, 80% untreated	FL, untreated
Source of tumor	FNA/core biopsy	FNA/core biopsy	Excisional biopsy
Idiotype	Recombinant	Recombinant	Hybridoma
Induction therapy	CVP (8 cycles every 3 weeks)	Rituximab (weekly ×4)	PACE/R-CHOP (6–8 cycles every 4 weeks)
Type of comparison (experimental/control)	2/1 randomization	1/1 randomization	2/1 randomization
Patient status before vaccination	First CR or PR	First CR, PR, or SD	First CR or CRu
Vaccination	Id-KLH + GM-CFSE or KLH + GM-CSF (sc, 7 doses)	Id-KLH + GM-CFSE or placebo + GM-CSF (sc, until PD)	Id-KLH + GM-CFSE or KLH + GM-CSF (sc, 5 doses)
Number of patients (actual/expected)	Vaccine: 192/240; control: 95/120	Vaccine: 174/171; control: 175/171	Vaccine: 76/250; control: 41/125
Primary end point	PFS (*p* < 0.01)	TTP (*p* < 0.01)	DFS (*p* < 0.01)
Results	Median PFS, 19.1 (experimental) versus 23.3 (control) mos (*p* = 0.297)	Median TTP, 9 (experimental) versus 12.6 (control) mos (*p* = 0.019)	Median DFS, 44.2 (experimental) versus 30.6 (control) mos (*p* = 0.045)
Reference	(49)	(50)	(51)

*CHOP, cyclophosphamide, doxorubicin, vincristine, and prednisone; CNOP, cyclophosphamide, mitoxantrone, vincristine, and prednisone; RTX, Rituximab; CRu, complete response unconfirmed; CVP, cyclophosphamide, vincristine, and prednisone; DFS, disease-free survival; GM-CSF, granulocyte-macrophage colony-stimulating factor; ITT, intent to treat; KLH, keyhole limpet hemocyanin; n.s, not significant; PACE, prednisone, doxorubicin, cyclophosphamide, and etoposide; PFS, progression-free survival; pts, patients; CR, complete response; PR, partial response; SD, stable disease; TTP, time to progression; mos, months*.

The Genitope trial enrolled 287 previously untreated patients with the aim to show a significant increase in disease-free survival (DFS) in the vaccinated cohort as its principal endpoint. Even though this was not achieved, among the vaccinated patients, those who mounted an anti-Id immune response experienced a significantly improved PFS, further strengthening the correlation between the induction of vaccine-specific immune effects and the clinical benefit (49). The Favrille study compared TTP between the vaccine and control cohorts, who included 349 patients in total, with ~80% being treatment-naïve, but failed to demonstrate any clinical improvement in the experimental arm (50). Unfortunately, immune responses were not monitored in these patients and the association between immunological and clinical effects could not be verified. The Biovest trial enrolled 234 previously untreated patients: 177 achieved a CR/CRu after induction chemotherapy and were thus randomized, but 60 of these patients did not receive the vaccine because of relapse or other reasons, thus missing the expected intention to treat (ITT) endpoint. However, among the treated patients, those who received the vaccine (*n* = 76) experienced a prolongation of the DFS by 13.6 months compared to those treated with the placebo (*n* = 41) (44.2 versus 30.6, *p* = 0.045), but without any increase in OS (51). In particular, treatment with Id of the IgM class, but not IgG, showed to significantly improve DFS compared to the isotype-matched control (52.9 versus 28.7 months; *p* = 0.001). Although results from the Biovest study are not definitive because of the non-met ITT and the low statistical significance level of the difference in DFS between the two cohorts, they granted BiovaxId the orphan drug status by the FDA. For a proper interpretation of this study, it is important to consider that patients who received the vaccine had to remain in remission during the period of the vaccine preparation. Since the average time of vaccine production was 8 months, it is possible that vaccinated patients had less aggressive and/or less chemoresistant diseases, thus explaining a longer-lasting complete response. Alternatively, these results may simply reflect the concept that complete tumor eradication predisposes to the achievement of a clinical benefit after vaccination.

Although the outcome of these phase-III clinical studies did not meet the high expectation, they have provided important information to improve the design of future trials. They indeed confirmed in significantly larger cohorts of patients (1) the safety and tolerability of Id-KLH produced either by recombinant or hybridoma technology; (2) the potential advantage of the latter method for the generation of more immunogenic and effective Id vaccines; and (3) the importance of inducing complete remission before vaccination in order to increase the probability of a clinical success. Moreover, results from these studies point to patients’ selection and vaccine formulation as the areas with the highest room for potential improvement, in particular in view of the better definition of the molecular prerequisites to achieve an effective antitumor immune response. Importantly, these findings may be useful to optimize the design of anti-lymphoma active immunotherapy across different types of vaccines.

## DNA-Based Vaccines

As an additional option to target DC *in vivo* and immunize cancer patients, viral vectors and plasmid DNA encoding TAAs have been exploited. This strategy requires *in vivo* transfection and antigen production. The optimized gene sequence is delivered intradermally, subcutaneously, or to the muscle, which allows, respectively, the *in vivo* transfection of professional APCs (epidermal keratinocytes and Langherans DCs) or myocytes and secondary cross-presentation of tumor antigens by the recruited DCs. The advantages of DNA-based vaccines over other immunization strategies include (1) the possibility of incorporating multiple epitope-encoding DNA regions to target several antigens in a single vaccine formulation, (2) no need to know the human leukocyte antigen (HLA) type because the protein products are processed *in vivo* by host APCs, (3) low production costs, and (4) the easy procedure required for their generation. However, as a drawback, it is possible that antigen expression, processing, and presentation take place in the improper cell subsets without the adequate stimuli, thus resulting in tolerance or an unwanted type of immunity rather than in the priming of an antitumor adaptive immune response (52).

Initial clinical trials demonstrated the feasibility and safety of vaccination with Id-encoding plasmid DNA, with no relevant levels of integration into host cellular DNA, or development of autoimmune reactions. However, due to the limited biological efficacy and no clinical benefit of Id-encoding naked DNA plasmid (53), more potent Id-DNA vaccines were generated by fusing the Id sequence to virus-derived immune stimulatory sequences (such as the fragment C of the tetanus toxin) (54) or cytokine-encoding genes (55), to favor DC chemotaxis, antigen uptake and presentation. In *in vivo* lymphoma models, these formulations showed prophylactic and therapeutic antitumor effects that relied on the induction of a specific T-cell response. As an additional strategy, pretreatment of the vaccination sites with low-dose cardiotoxin was found to generate a favorable immune microenvironment, which facilitated antigen-specific T-cell priming toward a long-term antitumor immunological memory (43).

The availability of more and more accurate mathematical algorithms for a better prediction of the most immunogenic peptides within TAAs will probably favor improving the design of DNA-based vaccines in the near future (56).

## DC-Based Vaccines

To overcome the limitation of producing a custom-made protein for each patient, targeting a single antigen, and relying on the host’s antigen processing machinery, presentation, and T-cell co-stimulation, loading DCs *ex vivo* with TAAs in the presence of the proper activation stimuli has also been exploited. In this case, DCs are properly differentiated from CD34^+^ hematopoietic progenitors or, more commonly, from peripheral blood monocytes in the presence of the proper DC differentiation and maturation cytokine cocktails, and pulsed with TAAs as to recapitulate *ex vivo* the early phase of DC activation. The source of DCs, TAAs, the antigen-engulfing strategy, cytokine cocktails, and the route of vaccine administration can be multiple and require precise consideration to optimize the therapeutic efficacy of DC-based vaccines (52).

Clinical efficacy of DC-based vaccines seems to be superior compared to that achieved by Id-protein vaccines against lymphoma (57), confirming observations in different tumor settings of the advantages of this strategy over protein-based vaccines (58). Interestingly, when a DC-vaccine was used to immunize against the single antigenic Id protein, FL patients with relapsed or residual diseases after induction therapy developed anti-Id T-cell and Ab responses associated with durable tumor regressions, in particular when Id was conjugated with KLH (59). DCs loaded with tumor cell lysates showed to elicit significant anti-lymphoma immunity in preclinical models (60) and in small clinical trials (61). In a pilot study, we showed that vaccination with autologous DCs loaded with apoptotic and necrotic autologous tumor cells increased natural killer (NK) cell activation, reduced Treg frequency and induced both T- and B-cell antitumor responses associated with clinical efficacy in 6 of 18 heavily pretreated indolent B-NHL patients with measurable disease (61). Interestingly, in responder patients, the humoral responses induced by vaccination were directed against common lymphoma antigens (62). Of note, we showed that the levels of immunogenic stimuli in dying lymphoma cells used to pulse DCs positively correlated with the probability of a clinical success of the vaccine (63). Therefore, favoring the occurrence of this process, namely immunogenic cell death (64), by exogenously supplying antigenic/proinflammatory signals to boost DC engulfing, cross-presentation, and maturation, may increase the efficacy of DC-based vaccines.

As additional modalities to load DCs *ex vivo* with the full lymphoma antigenic repertoire, fusion of DCs with tumor cells (65) and transduction of DCs with tumor-derived mRNA have started to be investigated in the preclinical setting (66–68). The latter is a promising technique in light of the minimal sample size required for the amplification of total tumor RNA, which considerably decreases the cost of vaccine production. However, it has to be considered that DC transduction channels TAAs primarily into the MHC-I presentation pathway, thus limiting the activation CD4^+^ T cells (69), which are crucial to sustain both Ab and CTL responses.

## *In Situ* Vaccination

The understanding that certain anticancer treatments, including antracyclines and radiation, can favor the induction of an immunogenic tumor cell death (64), supported the possibility to combine them with proper immune adjuvants to achieve *in vivo* a vaccinal antitumor effect (Figure 1). To facilitate *in vivo* TAA processing and T-cell cross-priming, toll-like receptor (TLR) agonists are particularly suitable as they can activate and bridge the innate and adaptive immunity (70). The preclinical observation that intratumoral injection of the TLR9 agonist CpG oligodeoxynucleotides plus systemic chemotherapy eradicated large tumors inspired the clinical evaluation of low-dose locoregional radiation plus intratumor CpG injection in low-grade B-NHL patients (71). This approach achieved clinical responses at distal tumor sites (abscopal effect) in association with the induction of tumor-reactive CD8^+^ T cells and reduction of intratumor Tregs (71). This study underscored the feasibility, safety, and efficacy to provide the conditions for the *in vivo* generation of an antitumor vaccine, thus overcoming the limitations for the manufacture of patient-specific products. Following these promising results, *in situ* vaccination with GpC and local radiation therapy was evaluated in resistant/refractory cutaneous-T-cell-lymphoma patients in a phase I/II study (72). Also in this case, treatment resulted safe and achieved systemic clinical responses, somehow associated with a positive immune modulation, in one-third of the patients. These findings point to the availability in the near future of a non-customized vaccine approach widely applicable with no requirement of any *ex vivo* cellular manipulation.

As a complementary modality to *in situ* vaccination, adoptive transfer of vaccine-primed autologous T cells after *in vitro* expansion, namely immunotransplant, has been exploited. Upon the achievement of the proof-of-concept in preclinical models (73), patients with newly diagnosed MCL were subjected to this procedure. In this case, the vaccine was made of autologous tumor cells that were treated *in vitro* with CpG and irradiated before administration into patients previously exposed to cytoreductive standard chemotherapy (74). Vaccine-primed T cells were then harvested, expanded *in vitro*, and reinfused after standard autologous stem cell transplantation. Preliminary results showed the feasibility of this approach in aggressive lymphoma patients and its efficacy in boosting antitumor T-cell responses. This provides the proof-of-principle for further investigations of the sequential combination of active and adoptive immunotherapy in cancer patients.

## T-Cell Therapies

The ultimate objective of active immunotherapy is to induce an endogenous immune response able to activate T cells against the tumor. The clinical experience with anti-lymphoma vaccines has clearly shown a limited efficacy of this strategy to consistently expand a sufficient number of activated antitumor T cells able to clear established tumors in pluritreated patients. With the same rationale of the use of immunotransplant, lymphoma patients can be adoptively transferred with an adequate amount of tumor-specific T cells optimized *ex vivo* to recognize and kill cancer cells, in order to maximize the probability to achieve a therapeutic effect (Figure 1). Two main T-cell therapeutic strategies have shown considerable success against B-cell lymphomas: transfer of EBV-specific T cells for the treatment of EBV-associated lymphomas and CAR T cells engineered to target B-cell lineage markers that continue to be expressed in the malignant clones.

Post-transplant lymphoproliferative diseases (PTLDs), caused by the reactivation of EBV infection in B cells of donor or recipient origin after allogeneic hematopietic stem cell transplantation (HSCT) or solid organ transplants (SOTs) respectively, continue to be a significant clinical problem (75). Management of heavily immunosuppressed patients with anticancer treatment poses several limitations, and standard treatment with rituximab eventually associated with less-intensive chemotherapy regimens often fail to cure PTLDs (76). EBV-infected B-cells do not actively produce virus, and, as such, are not sensitive to antiviral agents (77), but express viral latency-associated proteins, which may represent effective targets for immunotherapy. Depending on the type of latency of EBV, malignant B cells express more or less immunogenic EBV antigens. Tumors that arise in severely immunocompromised patients, such as in the early phases after allogenic HSCT or in SOT recipients, are usually highly immunogenic and express all the 10 EBV latency-associated proteins. Expressing the same 10 viral antigens and high levels of class-I and class-II HLA as well as co-stimulatory molecules, EBV-transformed B cells are optimal APCs for the activation of HLA-matched EBV-specific T cells to be used in this setting. With this strategy, polyclonal anti-EBV CTLs have been rapidly and abundantly generated from healthy EBV-seropositive donors, and proved safe and effective in preventing or treating PTLDs in recipients of allogenic HSCT (78, 79). Based on these encouraging results, a similar strategy has been attempted to treat post-SOT PTLDs. In this case, anti-EBV CTLs have been generated from the organ recipients and demonstrated some success in patients with either elevated EBV viral load or active disease (80–82). The constant immunosuppression status and the fact that SOT patients do not receive any lymphodepleting pre-conditioning treatment, which instead favors T-cell expansion in HSCT recipients, may account for the reduced persistence and efficacy of the transferred anti-EBV CTLs in this setting. However, these results have been crucial to demonstrate the feasibility of anti-EBV T-cell therapy in SOT recipients and the absence of any risk to induce rejection of the transplanted organ.

Interestingly, efficient control of PTLD was also achieved when “off-the-shelf” EBV-specific T cells derived from partially matched third-party donors were used in the context of both SOT and HSCT (79, 83, 84). This represented a dramatic improvement in the management of PTLD patients as anti-EBV CTLs of different HLA specificities may be generated and banked in advance in order to be readily available when needed. Very recently, anti-EBV CTLs derived from either patient’s transplant or third-party donors have shown similar substantial efficacy in producing long-lasting remissions in patients with aggressive rituximab-resistant post-HSCT PTLDs [(85), AACR Annual Meeting 2015, abstract CT107]. These results granted breakthrough therapy designation to anti-EBV CTLs generated from third-party donors for the treatment of patients with rituximab-refractory PTDLs.

Since EBV-related HLs and B-NHLs express only the weakly immunogenic EBV latency proteins (type II EBV latency proteins, LMP1, LMP2, and EBNA-1) (86), T cells specific for these antigens rather than polyclonal anti-EBV T cells need to be infused in order to achieve a clinical effect. However, the time required for their generation makes the procedure not suitable for the treatment of patients with active disease (87). For the same reason, T-cell therapy has not been developed for the treatment of the type-I EBV latency BL, which express only the poorly immunogenic protein EBNA1.

To broaden the specificity of T cells against multiple TAAs, transduction of high-affinity TCRs or CARs into mature or precursor T cells have been accomplished to make adoptive immunotherapy more easily available for patients with different tumor types (88). This latter option has found relatively wide application for the treatment of B-cell malignancies. CARs contain an extracellular domain with the Ab variable regions recognizing the target TAA genetically fused to the intracellular CD3ζchain (89). T cells transduced with CARs are therefore redirected toward the target antigen via the Ab regions, which, once engaged, trigger the CD3ζchain-downstream signaling cascade for T-cell activation. The activity of CAR-T cells thus becomes independent from HLA recognition, and this constitutes a major advantage of this strategy. The consistent expression of the B-cell lineage markers CD19 and CD20 across most B-cell malignancies and the reported safety/efficacy of anti-CD19/-CD20 mAbs in these diseases made them the preferred targets for CAR-T cells. In the preclinical setting, first generation CAR-T cells against CD19 or CD20 (CD19-/CD20-ζ) showed adequate engraftment and anti-lymphoma activity in either mice xenografted with patients’ tumors and autologous CAR-T cells or in syngeneic murine models following lymphodepletion with cyclophosphamide or radiation (90–93). However, the limited persistence of these CAR T-cells, partially driven by the presence of endogenous normal B cells expressing the target antigens (92, 94), led to the development of second-generation CARs, where the CD3ζ region was fused to the intracellular signaling domains of T-cell co-stimulatory molecules, such as CD28 or CD137 (4-1BB). CD19⋅CD28-ζ and CD19⋅CD137-ζCAR-T cells demonstrated enhanced functions, proliferation and survival, and resistance to Treg suppression, which resulted in increased persistence and antitumor activity in xenografted mice (95–99). This strategy seemed to be particularly effective when the tumor cells expressed low levels of ligands for co-stimulatory molecules (95, 97), because, being transduced with co-stimulatory domains, second-generation CAR-T cells did not depend anymore on physiologic co-stimulation signals. Third generation CAR-T cells with all the three T-cell signaling domains fused together (CD3ζ, CD28, and 4-1BB) have not definitely proven to exert a better antitumor activity (98, 100). Another approach studied with the aim to increase CAR-T cell *in vivo* persistence has been to engineer T cells specific for common viruses, such as EBV. Transduced virus-specific lymphocytes maintain the capability to become physiologically activated *in vivo* through their natural T-cell receptor and to persist in the memory compartment, offering the advantage to control their expansion by vaccination with the cognate viral antigens (101, 102).

Based on these preclinical findings, clinical studies mainly investigated second-generation CARs, either with CD28 or 4-1BB signaling domains, alone or in combination with lymphodepleting conditioning regimens. Experience accumulated so far in patients with B-cell malignancies indicates (1) the feasibility of generating and using CARs in the clinical setting, (2) the advantage of retro/lentiviral gene transduction methods over plasmid transfection technology to generate more functional CAR-T cells (no resistance selection genes, shorter culture periods), and (3) the importance of lymphodepleting pre-conditioning treatments to facilitate engraftment and in turn the therapeutic effects of CAR-T cells, with no specific restriction to the regimen to be applied. These observations, made initially in early small clinical trials with refractory/resistant B-NHL and acute lymphoid leukemia patients, are being confirmed in larger studies (39, 103–106). Persistent clinical responses and relatively manageable toxicities were induced by autologous CD19 CAR-T cells in patients relapsing after multiple lines of treatments. Interestingly, this approach proved effective also when donor-derived allogeneic CAR-T cells were administered in B-NHL patients who relapsed or were at high risk to relapse after allogeneic HSCT (107). Redirecting allogenic T cells against a TAA with CARs appeared an effective strategy to uncouple graft-versus-host-disease (GVHD) and graft-versus-leukemia (GVL) effect in patients who failed HSCT and DLI. In this context, the use of virus-specific T cells for the generation of donor-derived CD19 CAR-T cells showed promising results in controlling both the disease and viremia in patients with viral reactivation after allogeneic HSCT (108).

Altogether these findings indicate the substantial therapeutic potential of CAR-T cells against B-cell malignancies; however, this approach has still a wide margin for improvement, which mainly relies on the need for a better understanding of the biology of CAR-T cells, more robust biomarkers of clinical response and methods to reduce toxicity. Cytokine release syndrome and neurologic toxicities are not uncommon side effects of CAR-T cells. Therefore, there is a huge effort toward the understanding of how to control the functions of these T cells. Preclinical studies have investigated the potential to eliminate CAR-T cells in case of toxicity by co-transducing chemically inducible apoptosis-promoting fusion proteins, such as Fas and Caspase 9 (109), or targets of cell-depleting antibodies, such as CD20 or truncated epidermal growth factor receptor (110, 111). By eliminating CAR-T cells themselves, however, such strategies abolish both their side effects and therapeutic potential. As an alternative option, already tested in patients, blocking IL-6 receptor with the specific mAb tocilizumab has shown promising results in reversing cytokine release syndrome while sparing expansion and therapeutic effects of CAR-T cells (112, 113).

Finally, in light of the potential ability of tumor cells to escape CAR-T cell therapy, for example, by downregulating the expression of the targeted antigen (112), it is important to study strategies for counteracting such mechanisms. Toward this goal, CAR-T cells engineered to target multiple and/or alternative (114, 115) lymphoma antigens or their combination with other immunotherapeutic modalities are under investigation.

## Targeting the Immune Microenvironment in Lymphoma

In order to grow and progress in lymphoid organs, lymphomas need to subvert immunosurveillance while preserving the pro-lymphomagenic functions of nearby immune cells, thus becoming real parasites of the immune system. The prototype example of the role of the crosstalk with immune cells in the lymphoma microenvironment is HL, where the Hodgkin and Reed–Sternberg (HRS) tumor cells account only for 1% of the affected tissue, being the rest all inflammatory cells, which provide crucial interactions through CD80 and CD40/CD40L for HRS cell survival. In this case, mechanisms of immune evasion include polarization of infiltrating T cells toward a T helper 2/Treg phenotype through the release of IL-10 and TGF-beta, and inhibition of NK cells and CTLs via overexpression of FAS ligand and the ligands of the immune checkpoint receptor programed-death 1 (PD-1) (116). As an additional demonstration of the importance of the immune infiltrate in lymphoma development and progression, genetic and immunohistochemical signatures of non-tumor cells in the neoplastic tissue currently represent the best predictors for B-NHL patients’ prognosis (29, 117–120). These studies showed that a reduced survival and the risk of transformation of indolent B-NHLs are associated with the infiltration of specific immune cell subsets. In particular, lymphoma-associated macrophages (29), CD4^+^CD25^+^FOXP3^+^ Tregs (121) monocytic myeloid-derived suppressor cells (bearing a CD14^+^HLA-DR^low/−^ phenotype) (122, 123), and exhausted T cells expressing intermediate levels of PD-1 (124) have been all associated with a negative clinical impact in FL patients. The fact that immune cells are not usually targeted by conventional treatments may explain why, despite major therapeutic advances, indolent B-NHLs still remain incurable, underscoring the importance of modulating the microenvironment as a part of the lymphoma treatment.

Lately, several strategies able to modulate T-cell functions have become available, allowing preclinical and in some cases clinical evaluation of the anti-lymphoma effects of Tregs inhibition, promotion of T-cell co-stimulation, and inhibition of immune checkpoints. The IL-2-diptheria toxin fusion protein denileukin diftitox (ontak), the anti-CD25 mAb daclizumab, and anti-folate receptor 4 (FR4) mAbs have been studied to deplete Tregs. Agonist mAbs directed against the co-stimulatory molecules, OX40 (CD134), glucocorticoid-induced TNF-related protein (GITR), and 4-1BB (CD137), have been used to boost antitumor T-cell functions, whereas blocking mAbs for the co-inhibitory molecules cytotoxic T-lymphocyte-associated antigen 4 (CTLA-4) and PD-1 have been employed to prevent a negative regulation of tumor-specific T cells (Figure 1). In lymphoma preclinical models, T-cell modulation by anti-OX40, -GITR, -CD137, -CTLA-4, or -FR4 mAbs has shown to significantly improve the therapeutic efficacy of several immunotherapeutic modalities, including antitumor vaccination and mAb therapy (125–127). This evidence has led to the clinical evaluation of T-cell modulating agents for the treatment of these diseases. CTLA-4 or PD-1 blockade with the mAbs ipilimumab or pidilizumab, respectively resulted in safe and induced modest but occasionally long-lasting clinical responses in replapsed/refractory B-NHL patients evaluated in early-phase trials (128–130). Interestingly the combination of pidilizumab and rituximab was well tolerated and active in patients with rituximab-sensitive FL relapsed after 1–4 previous therapies (131), underscoring the importance of further investigating this strategy in B-NHL patients. An unexpected therapeutic activity of single-agent anti-PD-1 mAb nivolumab was instead found in heavily pretreated HL patients (40), which may thus provide a real therapeutic option for this patient category with an otherwise very unfavorable prognosis. The basis for the substantial clinical effects observed in this study probably relies on the high frequency of copy-number gain in PD-1 ligand loci in the enrolled patients (40). This points to a genetically defined sensitivity to PD-1 blockade in this disease. Given that genetic alterations of PD-1 ligands were not reported to be as frequent in newly diagnosed HL patients, it is possible that they define a subset of HLs with a particularly adverse prognosis.

Another straightforward way to redirect immune cells against lymphoma clones within the tumor microenvironment has been to modulate NK cell activity to enhance the effector functions of mAb therapy. As one of the major mechanisms of action of therapeutic mAbs is Ab-dependent cell mediated cytotoxicity (ADCC), whereby NK cells and phagocytes are redirected to the targeted tumor cells through Ab Fc receptors, the possibility to further co-stimulate ADCC cellular mediators via immunomodulatory mAbs was hypothesized to synergize with antitumor mAbs. Since the co-stimulatory molecule 4-1BB is upregulated on NK cells upon Fc receptor engagement (132), agonist anti-4-1BB mAbs have been investigated in combination with anti-lymphoma mAbs with the aim to increase antitumor ADCC. According to this hypothesis, agonist anti-4-1BB mAbs significantly improved the anti-lymphoma effects of anti-CD20 mAbs in preclinical models (127). In addition, human NK cells were found to consistently up-regulate 4-1BB when exposed to rituximab-coated autologous lymphoma cells (127), providing the rationale to explore the combination of anti-4-1BB and -CD20 mAbs in the clinical setting. Based on these findings, a phase-Ib study of the anti-4-1BB mAb urelumab and rituximab in relapsed/refractory B-NHL patients has recently started (NCT01775631).

Finally, because of their immunomodulatory properties, thalidomide and its derivatives have been also exploited to target the microenvironment in B-NHLs. Besides their potential to directly interfere with tumor growth and induce apoptosis in tumor cells, these agents promote antitumor immunity, including mAb-mediated ADCC, and antiangiogenic effects. Lenalidomide has been the most widely investigated drug in this category, showing significant single-agent anti-lymphoma activity in phase-II trials (133–136), in particular against aggressive B-NHLs. Building upon these results, a larger phase-II study was initiated to test safety and efficacy of lenalidomide in MCL patients relapsed after a second-line therapy with bortezomib, for whom no therapeutic options were available (137). Based on the tolerability and durable clinical responses induced by lenalidomide in this patient population, in June 2013, the FDA approved this drug for the treatment of MCL patients relapsed or progressed after two prior therapies including bortezomib. Lenalidomide has also been explored in combination with rituximab in relapsed/refractory indolent and aggressive B-NHLs showing significant and consistent clinical efficacy across different phase-II trials (138, 139). Interestingly, this combination compared favorably with single-agent rituximab in historical controls (5, 140). In light of the activity of lenalidomide against aggressive B-NHL, its combination with rituximab-based chemotherapy (CHOP, cyclophosphamide, doxorubicin, vincristine, and prednisone) has been investigated as front-line therapy for these diseases in phase-II studies, proving to be highly effective and safe also in this contest (141–143). A phase-III randomized, double blind, placebo-controlled, and multicenter study to compare the efficacy and safety of lenalidomide with R-CHOP versus placebo with R-CHOP in patients with previously untreated DLBCL is underway (NCT02285062).

## Conclusion

New curative treatments are needed for B-cell lymphomas. The availability of specific antigens and the easy accessibility of the immune system to these diseases have supported the extensive study of immunotherapy in the attempt of improving the management of B-cell lymphoma patients. Even though active immunotherapy through antitumor vaccination theoretically represents the ideal immunotherapeutic modality to induce antitumor immunity and control disease recurrences, the possibility to activate effective endogenous immune responses has proven challenging even in lymphoma patients. Alternative approaches to promote tumor targeting by T cells have more recently been investigated with promising results, with T-cell therapy regaining considerable attention thanks to the recent clinical successes of CAR-T cells. However, with the increasing use of anticancer immunotherapy, we are becoming aware of the advantages and limitations of the different strategies now available to activate/modulate antitumor immunity. It seems clear that if active and adoptive immunotherapy as well as immunomodulatory mAbs may not reach the desired activity as single agents, they can be exploited in rational combinations to maximize the probability of a clinical benefit (57). In conclusion, the significant advancements in the development and application of immunotherapy against B-cell lymphomas hold promise for a better definition of curative options for these diseases in the near future.

## Conflict of Interest Statement

The authors declare that the research was conducted in the absence of any commercial or financial relationships that could be construed as a potential conflict of interest.
